# Cosmeceutical Therapy: Engaging the Repercussions of UVR Photoaging on the Skin’s Circadian Rhythm

**DOI:** 10.3390/ijms23052884

**Published:** 2022-03-07

**Authors:** Camille Keisha Mahendra, Hooi-Leng Ser, Priyia Pusparajah, Thet Thet Htar, Lay-Hong Chuah, Wei Hsum Yap, Yin-Quan Tang, Gokhan Zengin, Siah Ying Tang, Wai Leng Lee, Kai Bin Liew, Long Chiau Ming, Bey Hing Goh

**Affiliations:** 1 Biofunctional Molecule Exploratory Research Group, School of Pharmacy, Monash University Malaysia, Bandar Sunway 47500, Malaysia; camille.mahendra@monash.edu (C.K.M.); thet.thet.htar@monash.edu (T.T.H.); alice.chuah@monash.edu (L.-H.C.); 2 Novel Bacteria and Drug Discovery Research Group, Microbiome and Bioresource Research Strength Jeffrey Cheah School of Medicine and Health Sciences, Monash University Malaysia, Bandar Sunway 47500, Malaysia; ser.hooileng@monash.edu or; 3 Medical Health and Translational Research Group, Jeffrey Cheah School of Medicine and Health Sciences, Monash University Malaysia, Bandar Sunway 47500, Malaysia; priyia.pusparajah@monash.edu; 4 School of Biosciences, Faculty of Health and Medical Sciences, Taylor’s University, Subang Jaya 47500, Malaysia; weihsum.yap@taylors.edu.my (W.H.Y.); yinquan.tang@taylors.edu.my (Y.-Q.T.); 5 Centre of Drug Discovery and Molecular Pharmacology (CDDMP), Faculty of Health and Medical Sciences, Taylor’s University, Subang Jaya 47500, Malaysia; 6 Physiology and Biochemistry Research Laboratory, Department of Biology, Science Faculty, Selcuk University, Konya 42130, Turkey; gokhanzengin@selcuk.edu.tr; 7 Chemical Engineering Discipline, School of Engineering, Monash University Malaysia, Bandar Sunway 47500, Malaysia; patrick.tang@monash.edu; 8 Advanced Engineering Platform, School of Engineering, Monash University Malaysia, Bandar Sunway 47500, Malaysia; 9 Tropical Medicine and Biology Platform, School of Science, Monash University Malaysia, Bandar Sunway 47500, Malaysia; 10 School of Science, Monash University Malaysia, Bandar Sunway 47500, Malaysia; lee.wai.leng@monash.edu; 11 Faculty of Pharmacy, University of Cyberjaya, Cyberjaya 63000, Malaysia; liewkaibin@cyberjaya.edu.my; 12 Pengiran Anak Puteri Rashidah Sa’adatul Bolkiah Institute of Health Sciences, Universiti Brunei Darussalam, Gadong BE1410, Brunei; 13 College of Pharmaceutical Sciences, Zhejiang University, Hangzhou 310058, China; 14 Health and Well-Being Cluster, Global Asia in the 21st Century (GA21) Platform, Monash University Malaysia, Bandar Sunway 47500, Malaysia

**Keywords:** ultraviolet rays, cosmetics, circadian rhythm, pigmentation, collagen degradation, DNA damage, apoptosis, natural product, melatonin, photoaging

## Abstract

Sunlight is an important factor in regulating the central circadian rhythm, including the modulation of our sleep/wake cycles. Sunlight had also been discovered to have a prominent influence on our skin’s circadian rhythm. Overexposure or prolonged exposure to the sun can cause skin photodamage, such as the formation of irregular pigmentation, collagen degradation, DNA damage, and even skin cancer. Hence, this review will be looking into the detrimental effects of sunlight on our skin, not only at the aspect of photoaging but also at its impact on the skin’s circadian rhythm. The growing market trend of natural-product-based cosmeceuticals as also caused us to question their potential to modulate the skin’s circadian rhythm. Questions about how the skin’s circadian rhythm could counteract photodamage and how best to maximize its biopotential will be discussed in this article. These discoveries regarding the skin’s circadian rhythm have opened up a completely new level of understanding of our skin’s molecular mechanism and may very well aid cosmeceutical companies, in the near future, to develop better products that not only suppress photoaging but remain effective and relevant throughout the day.

## 1. Introduction

The circadian rhythm is a 24 h periodic cycle that modulates the behavior and physiology of almost every living organism on Earth. Following environmental changes, the central regulator, residing in the suprachiasmatic nucleus (SCN) of the anterior hypothalamus, drives and synchronizes the circadian rhythm of peripheral tissues through neuronal and hormonal signals [1]. In a molecular context, core clock genes are responsible for the circadian rhythmic expression in mammals and these genes are the *circadian locomotor output kaput (CLOCK)*, *brain and muscle ARNT-like protein 1 (BMAL1)*, *periods (PER1, 2 and 3)*, and *cryptochromes (CRY1 and 2)*. Utilizing transcriptional–translational feedback loops to regulate the clock mechanism, CLOCK and BMAL1 protein initially form CLOCK: BMAL1 heterodimers to initiate the transcription of several genes with E-box cis-regulatory enhancer sequences. These include the transcription of *PER* and *CRY* genes. Both PER and CRY then form PER: CRY heterodimers that suppress the CLOCK: BMAL1 complex in the nucleus, negatively regulating its transcription [2]. Other than regulating *PER* and *CRY* genes, CLOCK: BMAL1 also activates the transcription of retinoic acid-related orphan nuclear receptors, *Rev-erbα* and *retinoic acid-related orphan nuclear receptor (Ror)α*. Both in turn then act as controllers of BMAL1 either by activating or repressing its transcription through their interaction with retinoic-acid-related orphan receptor response elements (ROREs) on the *BMAL1* promoter [2,3,4]. In addition, it had also been discovered that not only Rev-erbα and Rorα but all members of Rev-erb (α and β) and Ror (α, β and γ) can regulate the expression of *BMAL1* [5]. The discovery of these genes and proteins enables further understanding of how biological processes in humans are influenced by the circadian rhythm, particularly through the light–dark cycle and through sunlight.

Sunlight plays an essential role in maintaining our sleep/wake cycles through a delicate balance of melatonin and cortisol [6]. Not only that, it even has a direct impact on our physiological state. Volunteers, involved in a sun-tanning clinical study, had been declared to have improved mental state and appearance after being repeatedly exposed to ultraviolet rays (UVR) through sun-tanning [7]. Furthermore, UVR also has an essential role in modulating our body’s homeostasis through the cutaneous neuroendocrine system of the skin. One such example, is the formation of vitamin D through ultraviolet B (UVB) exposure on our skin, which in turn is known to modulate bone metabolism, mineral homeostasis, glucose homeostasis, etc. [8]. Vitamin D and its activated hydroxyderivatives also have antioxidant properties and the ability to attenuate UVB-induced DNA damage and skin inflammation, slowing skin aging [9,10]. Additionally, UVR exposure on the skin also affects the immune system and skin pigmentation through a system of neuropeptides and neurohormones [11,12,13,14,15]. As extensive discussion on this complex communication between UVR, the skin as a neuroendocrine system and the central circadian rhythm have been previously covered and will not be the focus of the current review. Nevertheless, the overexposure of UVR from the sun can still cause susceptible damage to the skin leading to skin photoaging. Evident symptoms of skin photoaging can be seen through the formation of irregular pigmentation, wrinkles, reduced skin elasticity, etc. [16]. In addition, prolonged exposure to sunlight even causes skin cancer as UVB can directly damage DNA by forming cyclobutene pyrimidine dimers (CPD) and pyrimidine-pyrimidone (6-4) photoproducts [17]. Besides that, the combination of both ultraviolet A (UVA) and UVB, were reported to induce oxidative stress and inflammation of the skin ultimately inciting the formation of irregular pigmentation and degradation of the skin’s extracellular matrixes [18,19].

In the past decade, the research question had slowly begun shifting to not only focus solely on UVR-induced skin photodamage but the scope had widened to include the effect UVR has on the skin’s circadian rhythm. This is because the skin was discovered to possess a multi-oscillatory system whereby different types of skin cells at different levels had autonomous oscillation that is fine-tuned to adapt to environmental changes which surpassed the common notion about the damage and regeneration concept in human skin that it predominantly servs as the first line of barrier against UVR assaults [20]. As a means to effectively protect the body against UVR exposure, the skin cells could function autonomously from the body’s central circadian rhythm. The overarching anti-phasic rhythm in the skin’s circadian rhythm mechanisms has been proven to exert regulation on cell division and DNA repair systems [6,21]. This complies with the preexisting studies, where night-shift workers demonstrated that the disruption of circadian rhythm imposes a decrease in DNA repair protein levels and increases the possibility of one developing cancer [6,22]. Therefore, it is imperative to study the influence UVR has on the skin’s circadian rhythm. This knowledge could also be translated into the preparation of cosmetic products dedicated for day or night usage, thus benefiting society. Taking into account the skin’s circadian rhythm, the effectiveness of these products could potentially be enhanced if proper understanding and synchronization were done following the skin’s oscillating rhythm. Given the importance and potentially significant impact on the cosmeceutical industry, the connection between UVR, the skin’s circadian rhythm, and photoaging indicate that it would be worthwhile to study these factors. Along the way, by tapping into mother nature, a rich source of chemical leads, and the structural diversity of natural active ingredients, researchers may ultimately discover some useful compounds that could modulate the skin’s circadian rhythm.

## 2. The Dance between Core Clock Genes and Solar Erythema

The three types of UVR that are produced by the sun are UVA, UVB, and ultraviolet C (UVC). UVA with the longest wavelength (320–400 nm) has a penetration ability up to the dermal level, while UVB (280–320 nm) only penetrates the epidermal layer. UVC (100–280 nm), on the other hand, does not penetrate through the ozone layer [23,24]. Despite the importance of sunlight as the main oscillator of our circadian rhythm, several studies have shown that acute and cumulative exposure to UVR disrupts the natural rhythm of the skin. As shown in Table 1, core clock genes were discovered to experience downregulation or upregulation depending on the type of UVR exposure. Different types of cells, such as keratinocytes and melanocytes, from both humans and mice, are shown in Table 1.

### 2.1. The Effect of NB-UVB BB-UVB on the Circadian Rhythm

NB-UVB is a common UVB lamp that is used in the treatment of psoriasis, atopic dermatitis, and vitiligo [30,31]. It emits only or mainly a single wavelength, which is ~311 nm and is mostly considered more efficacious in the treatment of some skin diseases [32]. The dosage required to cause damage is also significantly higher as compared to BB-UVB [33]. In the study done by Nikkola, et al. [34], participants with increased expression of CRY2 presented higher sensitivity to NB-UVB exposure compared to those with a lower expression of CRY2. Later experimentations on the epidermis and dermis layers of adult skin type II and III biopsies then demonstrated significant downregulation of *CRY2* after exposure to NB-UVB. In comparison to *CRY2*, *CRY1* only experienced a slight increase in expression 24 h after irradiation, giving rise to a significant *CRY1/2* ratio [25]. The mention of this ratio is important as a significant *CRY1/2* ratio is an indicator of the occurrence of DNA damage given that *CRY2^−/−^* cells have a higher propensity to elevate DNA damage accumulation [35]. Therefore, based on these experiments, it can be seen that prolonged exposure to NB-UVB actively disrupts the expression of *CRY2* inducing higher chances of one accumulating DNA damage and ultimately skin cancer. There could also be a possible connection between *CRY2* levels and sensitivity to NB-UVB, however, additional studies encompassing a larger population pool have to be conducted to substantiate this hypothesis.

Similarly, studies on BB-UVB also exhibited significant dysregulation of other clock genes, *BMAL1*, *CLOCK* and *PER1*, in epidermal cells (Table 1). These were also accompanied by an increase in DNA damage markers and activation of apoptosis pathways. When HaCaT cells were irradiated by 12.5 mJ/cm^2^ BB-UVB, *miR-142-3p* microRNA was increased, which in turn suppressed the expression of *BMAL1* mRNA and protein, leading to an increase in DNA damage [28]. In support of this finding, HaCaT cells irradiated with 5 mJ/cm^2^ BB-UVB also elevated the expression of phosphorylated p53, checkpoint kinase 1 (CHK1), and p21 [26]. An increase in *p53-upregulated modulator of apoptosis (PUMA)* and poly (ADP-ribose) polymerase (PARP) cleavage was also observed, indicating the potential activation of intrinsic apoptosis via caspase 3 [26,36]. Moreover, data attained from flow cytometry studies even showed that many HaCaT cells began entering phases of early and late apoptosis after exposure [26]. However, when *BMAL1* and *CLOCK* were silenced (separately), cell proliferation in sham-irradiated HaCaT cells was increased. In contrast to normal irradiated HaCaT cells, both *BMAL1*- and *CLOCK*-silenced cells also suppressed the number of cells in the late apoptosis phase and PARP cleavage. The expression of *PUMA*, p21, and DNA-damage marker γ-H2A histone family member X (H2AX) were also decreased compared to normal irradiated HaCaT. Conversely, the level of phosphorylated p53 and CHK1 in irradiated cells was not affected when BMAL1 and CLOCK were silenced. Thus, the authors suggest that BMAL1 and CLOCK do play a role in controlling cell apoptosis and DNA damage but probably at later stages of the ataxia telangiectasia and Rad3-related protein (ATR)–CHK1–p53 pathway in HaCaT cells [26]. As HaCaT cells are immortalized cell lines, the authors proceeded to compare the results obtained with HKC cells HKC. With this cell line, they discovered that no increase in cell proliferation was seen after *BMAL1* and *CLOCK* silencing. Additionally, *CLOCK*-silenced HKCs experience a decrease in p53 levels compared to normal irradiated HKCs [26]. This shows that in primary keratinocyte cells, *CLOCK* can affect the expression of p53 and it could be possible that the point mutations in the *p53* gene of HaCaT affects its interaction with CLOCK [37]. However, this possibility needs further confirmation. Other than that, the connection between clock genes and p53 under UVR conditions should also be further investigated as p53 was formerly reported to be able to modulate the circadian rhythm by repressing the expression of *PER2* under genotoxic stress. This was done through the binding of p53 to the *PER2* promoter, effectively blocking the attachment of BMAL1: CLOCK to the promoter and its transcription [38]. The protein expression of p53 and mouse double minute 2 (mdm2) were also reported to have rhythms that were anti-phasic to each other, confirming that p53 does have a role in the circadian rhythm. Subsequently, the phosphorylation rate of CHK1 was also demonstrated to change with time of day in mice skin post UVB exposure [39]. Therefore, it would be interesting to uncover the dynamics of the relationship between *CLOCK*, *PER2*, and p53-involved pathways in the skin’s circadian rhythm under the effect of UVR exposure. Finally, there was an increase in keratin 1 and 10 proteins when *BMAL1* and *CLOCK* were silenced, indicating that the cells actively enter stages of cell differentiation when *BMAL1* and *CLOCK* expressions are dysregulated [26].

### 2.2. Rhythms in Animals and Humans: How Do We Maximize Our Cosmetic Product?

**Figure 1:**
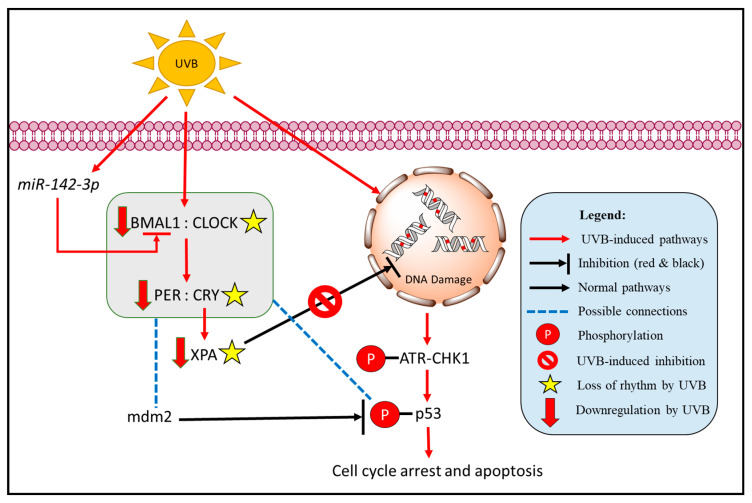
The effect of UVB on core clock genes and its interconnected pathways in cell-cycle arrest and cell apoptosis.

By connecting the dots, the various connections between core clock genes and UVB-induced DNA damage and cell apoptosis are summarized in Figure 1. As depicted, UVB exposure brings about the suppression of core clock genes, preventing the activation of DNA repair. At the same time, it directly causes DNA damage and incites cell-cycle arrest through the phosphorylation of p53, eventually leading to cell apoptosis. This figure was also drawn based on in vivo studies on mice. Considering an organism as a whole, in vivo studies using mice demonstrated that the regulation of cell division and oxidative phosphorylation of mice skin are anti-phasic, whereby genes related to the cell division process peak in the early hours of the morning (4 am) while those of the oxidative phosphorylation process generally peak around late afternoon (4 pm). Further experimentation then showed that mice skin had even higher amounts of DNA damage in the morning (2 am) as compared to when the skin was irradiated in the afternoon (2 pm). However, in *BMAL1^−/−^* mice, not only were the anti-phasic balance between cellular division and oxidative phosphorylation were lost, but there was also no longer any distinction in the amount of DNA damage between the morning (2 am) and afternoon (2 pm) samples [40]. Comparably, the same effect was also seen in *CRY1^−/−^*, *CRY2^−/−^* mice, where not only were there no longer circadian rhythmicity to DNA replication but the rhythmic expression of xeroderma pigmentosum group A (XPA) was lost as well [21]. The loss of both XPA rhythm and expression is detrimental indeed to the repair of DNA damage as XPA is a core factor involved in the nucleotide excision repair (NER) pathway and it is through this pathway that DNA adducts formed by UVR irradiation were repaired [41]. In mice, the expression of XPA is anti-phasic both to CRY1 and the rhythm of DNA synthesis [21]. Due to its rhythmic nature, the repair rate of CPD and pyrimidine-pyrimidone (6-4) photoproducts also follows suit. Mice skin that was exposed to UVB were able to repair CPD and pyrimidine-pyrimidone (6-4) photoproducts faster during the late afternoon hours (4 pm) compared to early morning (4 am), according to the rhythmic expression of XPA. This is even more evident with the rate of repair for CPD as its repair was observed to stop completely in the early morning, when the XPA level was the lowest, only to resume in the late afternoon hours after XPA levels begin peaking [21]. As well as affecting the DNA repair system, the loss of *CRY1/2* and *PER1/2* also equalized the number of apoptotic cells in the skin of mice from both irradiated morning (4 am) and late afternoon (4 pm) groups [39]. This could be due to the loss of rhythmicity in the regulation of cellular apoptosis pathways as the expression of CHK1, p53, p21, and mdm2 are controlled by clock genes [39]. Therefore, according to these studies, it is implied that (1) any disruption in the circadian rhythmicity of *BMAL1*, *CRY*, and *PER* could potentially lead to dysfunction in DNA repair and induction of cell apoptosis in badly damaged cells, leading to an increase in the potential of developing skin cancer; and (2) skin exposure at certain times of day is more damaging than others and therefore, it is important to avoid exposure at certain times according to the skin’s rhythmicity. As predicted, SKH-1 mice that were irradiated continuously in the morning for 25 weeks developed bigger and more invasive tumors compared to those that were exposed in the evening [21].

**Figure 2:**
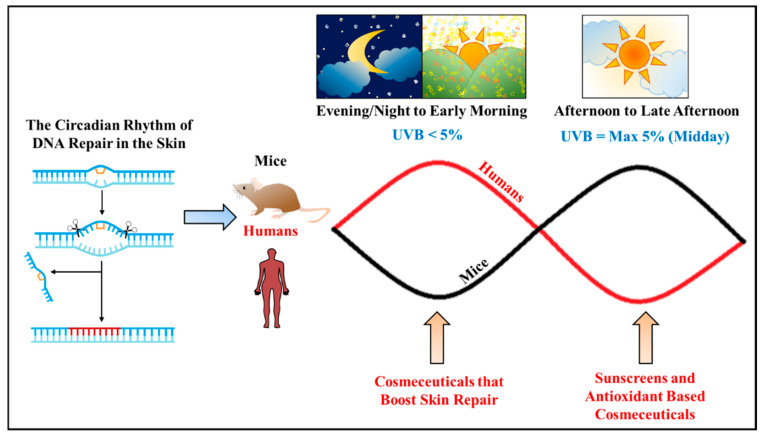
The circadian rhythm of DNA repair in the skin of both humans and mice, and the use of various cosmetic products according to the circadian rhythm to maximize its efficacy against photoaging. The red line represents the circadian rhythm for DNA repair in human skin while the black line represents that of mice skin. An opposite rhythm was observed for mice and humans indicating that humans are more sensitive to UVR-induced DNA damage during midday to late afternoon compared to being exposed in the morning.

Nonetheless, as humans are diurnal by nature it is suspected that our core circadian clock genes and its downstream genes are expressed in an opposite phase to those of nocturnal rodents. In short, it was hypothesized that our key repair genes are mainly expressed in the morning instead of in the afternoon/late afternoon, thus, making the morning sun less “toxic” compared to afternoon/late afternoon sun [21]. This hypothesis is supported by a study where participants demonstrated a higher erythema index (EI) when they were irradiated in the evening as compared to the morning hours [34]. On the other hand, in another study, the expression of several key repair genes, such as *XPA*, *RAD50*, and *PARP1* within human leukocytes, was observed to increase in the early hours of the morning, dip when approaching the afternoon to late afternoon hours, and then increase again slowly during the night to peak once again in the morning [22]. Thus, it can be inferred that DNA repair in humans is most likely to occur during the night and morning hours before being suppressed in the afternoon to late afternoon hours, which is the complete opposite to mice, as depicted in Figure 2. It is interesting, however, that the human body would suppress DNA repair in the afternoon when the penetration level of UVB through the ozone is at its peak and is most intense, causing the most DNA damage [42]. Therefore, following the rhythm of our skin’s DNA repair, cosmetic products such as night creams could be better designed to maximize repair rates during the night and morning. Conversely, products containing sunscreen and antioxidant properties should be used in the afternoon when photodamage is at its peak and our skin is at the weakest in repairing UVR-induced DNA damage. This is to protect the skin and at the same time reduce the production of reactive oxygen species (ROS) in the skin, suppressing skin inflammation, collagen degradation, and irregular pigment formation—all of which are hallmarks of photoaging. In addition, active ingredients, other than sunscreens, that can protect the natural rhythm of core clock genes from UVR should also be researched to benefit consumers in decreasing the rate of photoaging. In general, there is much benefit to considering our skin’s circadian rhythm in the manufacturing of cosmetic products. Not only would product efficacy be increased but there would also be a form of precision therapy that is currently lacking in the field of cosmetic science.

## 3. The Role of the Circadian Rhythm in Skin Pigmentation

As irregular pigmentation is often thought to be undesirable, it is critical to look into the mechanism behind UVR’s impact on the circadian rhythm and how it induces melanogenesis. Firstly, the distribution of melanin generated by melanocytes to its surrounding keratinocytes gives rise to our skin pigmentation. Located at the dermal/epidermal border, melanocytes utilize elongated dendrites to transfer melanosomes containing melanin to keratinocytes. This is then carried by keratinocytes at the basal layer towards the surface of our skin as new cells push them upwards to the upper layers [43]. There are two types of pigmentation that make up our skin coloration—eumelanin and pheomelanin [44]. Subsequently, it is three enzymes in the tyrosinase family—tyrosinase (Tyr), tyrosinase-related protein 1 (Tyrp1), and tyrosinase-related protein (Tyrp2)—that are responsible for the biosynthesis of both pigments [45]. Nevertheless, the determining factor for skin pigmentation formation is the signals produced by opsins. Opsins are members of the heptahelical G protein-coupled receptors (GPCRs) that are sensitive to light. Primarily, opsins serve as photosensors in a mechanism known as phototransduction, where photons from the environment are absorbed and transformed into cellular responses. There are five subfamilies of opsins—cone opsin (OPN1), rhodopsin (OPN2), encephalopsin or panopsin (OPN3), melanopsin (OPN4), and neuropsin (OPN5) [46]. OPN1 and OPN2 can be found in the retina and have a role to play in daylight and twilight vision, respectively [47]. OPN3, on the other hand, can regulate pigmentation under the induction of blue light [48]. OPN4 and OPN5 are instead involved in circadian photoentrainment of SCN [49,50].

When melanocyte cells were irradiated with UVA, the expression of *OPN2*, *OPN4*, *CLOCK*, *BMAL1*, *PER1*, and *XPA* was increased in murine Melan-A melanocytes and B16-F10 melanoma. Expectedly, the production of melanin also immediately increased after UVA exposure [51]. Conversely, the increase of *PER1* after 4.44 kJ/m^2^ UVA did not occur again in the later study done by de Assis, et al. [52]. After exposing the same cell lines to the same dosage three times cumulatively, the expression of *PER1* was immediately repressed instead by UVA, even though the impact of UVA did slowly lose its effect after repeated irradiation [52]. Reasons for this could be due to the difference in timing of when the sample was being studied after UVA exposure. After UVA exposure, a clear shift in the *PER1* circadian rhythm was observed, whereby the unexposed cells experienced a decrease in *PER1* while the exposed cells were still in the process of reestablishing balance for the sudden loss in PER1 expression. Nevertheless, the association between *Per1* suppression and increased melanin production is supported by the study done by Hardman, et al. [53]. Through silencing of both *PER1* and *BMAL1*, melanogenic activity within human epidermal melanocyte and hair follicles was activated, suggesting that suppression of both these core clock genes has a direct impact on melanogenesis [53]. On the other hand, Sarkar, et al. [54] instead demonstrated that overexpression of BMAL1 increases melanin levels by binding to the E′-box region within the *microphthalmia-associated transcription factor (MITF)* promoter, in an attempt to reduce DNA damage caused by UVB radiation. In fact, the circadian rhythmicity of *MITF* was in-phase with *BMAL1*. Additionally, they also proved that, in sync with the increase of BMAL1, *PER2* was decreased in the formation of melanin [54]. This discrepancy in data could be due to the difference in cell lines and species used across these studies. Another reason could also be that UVA and UVB might involve different pathways that modulate the expression of core clock genes, leading to the difference seen between studies. Finally, it is also important to continuously analyze the expression of core clock genes to show their rhythmic expression, the impact UVR has on these genes upon irradiation, and the time required to re-stabilize their rhythm. These are critical observations that studies using singular pinpointed hours could not achieve but instead would create discrepancies in the data in comparison with other studies as the clock genes could be in any stage of the recovery phase (either upregulated or downregulated to recover their original rhythm). Occasionally, complete shifts (by hours) of the rhythm were even observed after UVR irradiation, hence, it is better to have a complete study of expression changes over longer periods of time [29]. Overall, it is still undeniable that UVA initiates the formation of melanin.

Next, OPN4 was attested to be essential to the expression of PER1 in melanocytes. Although OPN2 is also involved in the formation of melanin, its exact interaction with the core clock genes under the influence of UVR during the melanogenesis process was still not fully elucidated [51,55,56]. The same could also be said for OPN3 [57,58]. Rather, silencing of *OPN4* initiated a completely opposite effect on *PER1* during UVA exposure. Instead of repressing *PER1* expression, *OPN4*-silenced cells experienced a sharp increase in *PER1* expression after irradiation. Furthermore, the response of *PER1* towards UVA exposure did not decrease with cumulative irradiation, as mentioned previously [52]. This means that *PER1* will experience dysrhythmia in its rhythm when *OPN4* is non-functional. Besides affecting the expression of *PER1*, the loss of *OPN4* also attenuated UVA-induced DNA damage, cell-cycle arrest, and cell apoptosis [52]. This indicates that the presence of OPN4 is important in reflecting the damage caused by UVA. It is most likely that OPN4 initiates these processes within the cells in response to UVA exposure. Subsequently, bioinformatic analysis found a negative correlation between OPN4 expression and the progression of human melanoma. As the disease progresses, *OPN4* expression was slowly lost while *BMAL1* was increased [52]. This suggests that without OPN4, there could be a higher risk for an individual to develop skin melanoma, or even worsen the disease progression. Thus, this confirms the importance of OPN4 as a photosensor and controller of our skin’s circadian rhythm in the response to UVA. Besides OPN4, OPN5 can also induce melanogenesis under the exposure of UVA and UVB through the calcium-dependent G protein-coupled and protein kinase C signaling pathways. When *OPN5* was silenced, MITF, Tyr, Tyrp1, and Tyrp2 were significantly downregulated as was the melanin content in human epidermal melanocytes [59]. OPN5 is not only an important factor in UVA photoentrainment in the retina but its presence in the skin can even influence the entire central circadian rhythm via its control of the core clock genes [60,61]. In mice, the expression of *OPN5* had been reported to be a necessity for the generation of full-amplitude diurnal rhythmic of *PER* gene expression in light and dark cycles [61]. Hence, it would be interesting to study how skin OPN5 and core clock genes interact under the exposure of UVR in the melanogenesis process.

In short, opsins play an important part in the melanogenesis process by modulating the core clock genes. However, current studies on the function of opsin in the skin circadian rhythm are limited. Questions such as the function of each opsin in UVR-induced melanogenesis and the propensity of the melanogenesis process occurring during the early morning and late evenings had yet to be answered. In addition, melanocytes are capable of producing neurotransmitters such as acetylcholine, opioids, and catecholamines [62]. Apart from that, various hormones such as α-melanocyte-stimulating hormone (α-MSH), proopiomelanocortin, and adrenocorticotropic hormone (ACTH), are also generated by melanocytes [62]. Working together, these neurotransmitters and hormones have a profound effect on the circadian rhythm [63,64,65]. Hence, it would only be right to question how the opsins in our skin and the neuroendocrine components of melanocyte interact with one another. However, as yet, there are still no substantial data collected to demonstrate their interactions. Therefore, it is clear that there still exists a large research gap on the study of opsins in connection to the skin circadian rhythm and its impact on the skin’s condition.

## 4. Loss of Circadian Rhythm alongside Skin Elasticity and Collagen Degradation

Another hallmark of photoaging is the formation of wrinkles and sagging skin [66]. This is also known as ‘solar elastosis’, whereby it describes the histopathologic changes occurring as elastic tissue in the dermal layer degenerates due to prolonged exposure to the sun [67]. The occurrence of this is due to the cumulative exposure of our skin to UVR which increases the production of matrix metalloproteinases (MMPs) that degrade the extracellular matrix (ECM) proteins in our skin. It is this imbalance of synthesis and degradation of ECM that slowly leads to the loss of our skin’s elasticity. [68].

### 4.1. Rhythmic Expressions of TIMP3 and AQP3

Research by Park, et al. [69] on the rhythmic changes of *aquaporin 3 (AQP3)* and tissue inhibitor of *metalloproteinase (TIMP) 3* proved to be interesting. Under the influence of UVB, core clock genes, such as *CLOCK* and *BMAL1*, were immediately downregulated in NHEK cells. Although *CLOCK* was able to resync back to its original rhythmic pattern, *BMAL1* experienced a phase shift [29]. This downregulation of clock genes was also followed by the suppression of both *AQP3* and *TIMP3* after exposure of UVB [69]. With the decrease of *TIMP3*, tumour necrosis factor (TNF)-α and MMP1 were also upregulated. Additionally, CCAAT-enhancer binding protein α (C/EBPα), chemokine (C-X-C motif ligand 1 (CXCL1), and interleukin (IL)-8 were also upregulated with the suppression of *TIMP3* [29]. Further knockout of the *CLOCK* gene then showed an instant loss of *TIMP3* rhythmic pattern, suggesting that there is a connection between TIMP3 expression with core clock genes [29]. There is also a possibility of an involvement of PER3 when the cells were exposed to UVB. This is because *PER3* suppression had been linked with the upregulation of MMP1 [70]. Perhaps under UVB exposure, CLOCK expression is reduced, affecting PER3 expression, and therefore, TIMP3 is suppressed leaving MMP1 to upregulate unchecked.

Unlike TIMP3, AQP3 had previously been discovered to have a rhythmic expression in accordance with core clock gene expression through the study conducted by Matsunaga, et al. [71]. Their study showcased how mice with mutated *CLOCK* gene experience a loss of *AQP3* rhythmicity, which in turn affects the hydration of the stratum corneum. The same was seen as well in HaCaT cells where CLOCK: BMAL1 heterodimer regulated the transcription of *AQP3* gene and silencing of the *CLOCK* gene affected both the production of *AQP3* mRNA and glycerol uptake [71]. As an aquaglyceroporin channel that transports glycerol and water molecules in the basal layer of the skin epidermis, the effect of AQP3 on skin elasticity is important. Mice with *AQP3* deficiency not only had decreased stratum corneum hydration due to the inability to withhold water in the epidermis but also reduced skin elasticity and delayed barrier recovery [72,73]. *AQP3* is also actively involved in the skin wound healing process by facilitating cell proliferation and migration [74]. On the other hand, the connection between TIMP3 and core clock genes as CLOCK-dependent diurnal genes has only recently been discovered, where *TIMP3* shares the same rhythmic pattern as *PER1* and *AQP3* [69]. TIMP3 is a member of the TIMP family that functions as inhibitors to MMP that is responsible for the development and homeostatic remodeling of our body’s extracellular matrix [75]. Previously, TIMP3 was reported to be an important factor in many diseases such as myocardial infarction, interstitial nephritis and fibrosis, and atherosclerosis [76,77,78]. One well-known role of TIMP3 is the modulation of the inflammatory pathways via TNF-α through TNF-α-converting enzyme (ADAM17) [79]. As the production of TNF-α under UV exposure induces the release of cytokines, bringing about dermal inflammation and eventually collagen degradation, control of TIMP3 becomes essential in inhibiting photoaging [18,80]. Therefore, the decrease of both AQP3 and TIMP3 by UVB through core clock genes demonstrates the effect UVB has on the skin’s circadian rhythm and its induction of skin photoaging. It would be interesting, however, if future research could showcase the changes in AQP3 and TIMP3 at different times of the day according to their rhythmicity. This is suggested as it could potentially help cosmeceutical companies better categorize their products and increase product efficacy, following the changes in our skin circadian rhythm.

### 4.2. Role of OPN3 in Collagen Degradation

UVA exposure on human dermal fibroblast was found to induce the expression of OPN3. This increase in OPN3 came with an increase of TIMP1 and expression of various MMPs (MMP 1, 2, 3, and 9). This took place through the activation of the calcium-dependent G protein-coupled signaling pathway, which phosphorylates Ca^2+^/calmodulin-dependent protein kinase (CAMK)II, cyclic adenosine monophosphate response element binding (CREB), p38, c-Jun *N*-terminal kinase (JNK), and extracellular signal-regulated kinase (ERK), leading to the expression of MMPs [81]. This is the first instance where OPN3 had been linked with collagen degradation markers in dermal fibroblast cells. Thus, being one of the photosensors in the photoentrainment of the circadian rhythm, further studies on how OPN3 and the core clock genes interact under UVA exposure is warranted [82]. This also includes the other opsins as they might be involved in collagen degradation.

## 5. Limitations and Research Gap in the Study of Skin Circadian Rhythm

**Figure 3:**
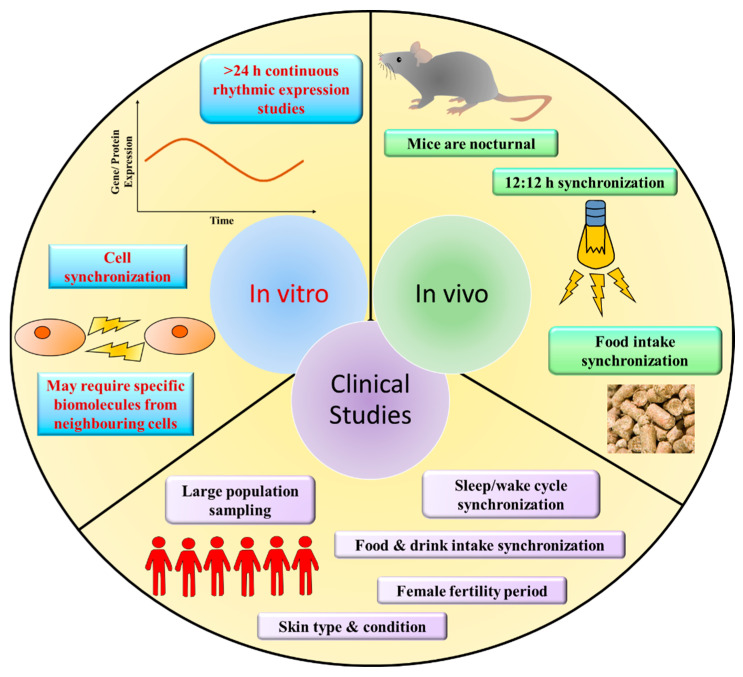
The important aspects to take note of during in vitro, in vivo, and clinical research when conducting a study on the skin’s circadian rhythm. At each level, there are different factors to consider to synchronize the samples that are most vital in circadian rhythm research.

When it comes to the skin’s circadian rhythm, there still exists a huge research gap and a lack of understanding of its mechanism and the impact it has on photoaging. Based on our understanding and perspective from large amount of research conducted, we propose that several reasons impede the study on the skin’s circadian rhythm (Figure 3). Firstly, in terms of in vitro experiments, there are some levels of difficulty in cell synchronization and the time required in conducting in vitro experiments. Not only must cells be well synchronized, but the study must be done over a continuous extended period to observe the rise and fall of gene expression. As mentioned earlier, a singular time point could very well produce contradictory data, creating a false sense of significant data. This is especially true when the clock genes themselves are dysregulated and are in the process of recovery after UVR exposure. By studying gene expression over a continuous period, the effect on core clock genes of UVR and its reversal via treatment can be observed clearly. However, due to the need for extended periods of study and 24/7 monitoring of cell changes, financial and technological requirements become an aspect to consider. Secondly, some cells even require the addition of certain biomolecules that are generated by their neighboring cells to activate certain pathways. For example, OPN5 requires the presence of all-trans retinal chromophores to reconstruct pigment [59]. Hence, there are certain limitations when it comes to monolayer in vitro cell culture studies. Therefore, some research turned towards the use of mice in the study of the skin’s circadian rhythm. Synchronization of mouse circadian rhythms can be controlled through the 12:12 h light–dark cycle but researchers have to be careful in monitoring their feeding time as it can induce changes in the mouse’s circadian rhythm [83]. There is also the issue of mice being nocturnal by nature, which is the exact opposite of humans, although a certain amount of difference between both species should be expected. This then brings us to clinical studies of the skin’s circadian rhythm in humans. There are many different factors to consider when it comes to the study of humans: (1) There had to be a significant number of participants in each study. This is because each person is different genetically, physiologically, mentally, and in their lifestyle, etc. Therefore, population study becomes helpful in removing outliers and obtaining the general norm among different individuals. (2) Skin condition and type (color) has also to be taken note of. (3) Synchronization of the sleep/wake cycle, food and drink intake, and for females, fertility period during the study has to be monitored as well. In the study done by Manzella, et al. [6], only females at early follicular phase were included in their study. Without all these factors being taken into consideration, the risk of obtaining discrepant data increases.

Despite that, several studies prove interesting in the field of cosmetics. One is the study conducted regarding the skin’s autophagy. Autophagy is a homeostatic cellular process that functions to either remove unwanted/damaged biomaterials or recycle cytoplasmic contents to promote cell adaption and survival under stressful conditions [84]. However, with age, autophagic activity in our skin has been shown to decrease. Comparing the microtubule-associated protein 1 light chain-3B (LC3B), a microtubule protein that is part of the autophagic process, expression in the synchronized primary skin fibroblast of a 2-day-old and a 67-year-old, the level of LC3B was significantly lower in adult cells. Unexpectedly, the expression of *LC3B* has a similar rhythmic pattern to the expression of *PER2* and autophagic expression was shown to peak at night [85]. This could imply that autophagic activity may have rhythmic changes following the expression of core clock genes. Further analysis, then showcased how 10 J/cm^2^ UVA was able to revive the lack of autophagy activity in older fibroblast cells and shift the expression of *LC3B* an hour earlier as compared to the young fibroblast cells [85]. It could be possible that certain amounts of UVA exposure could aid in reviving the autophagic functions of older cells but this would require further studies on this subject. In addition, other studies on proteins, such as *Krüppel-like factor (KLF) 9*, peroxiredoxin 2, and *sirtuin (SIRT) 3* and *4*, also showed that they all have rhythmic patterns in their expression in the human epidermal keratinocytes [86,87,88]. KLF9, a member of the KLF family, was suggested to affect cell proliferation and differentiation, while peroxiredoxins are antioxidant enzymes that are suppressed by UVB radiation [18,86,89]. As for SIRTs, they are ADP-ribosyltransferases and NAD (+)-dependent deacetylases that are involved in cell metabolism and epigenetics [87]. Hence, based on the few examples of these proteins, it can be seen that there are potentially many other proteins, either under the control of or perhaps in control of the skin’s circadian rhythm, that have yet to be studied in detail. As a matter of fact, the circadian involvement in UVB-induced inflammation has also not been well looked into. Other than the study done on mice in which inflammatory markers were seen elevated more in the morning than in the evening, the connection between the circadian rhythm and inflammatory pathways was mostly researched in diseases such as influenza and atherosclerosis [39,90,91]. Thus, this is another research gap, which is vital in the study of photoaging that had to yet to be breached. Finally, it is highly important for future cosmetic development to consider tailor-making products following the skin’s circadian rhythm. This is because not only does this increase product efficacy and desired outcomes but the mere idea of it is a door to an entirely new profitable marketing strategy in the cosmetic business.

## 6. Melatonin and Natural Products as Cosmeceutical Agents

The use of cosmetic products in beauty enhancement had been going on for centuries, dating back to 100,000 BC where the Neanderthals used body paints of mud and plants to decorate their bodies. Later, Egyptians utilized “cosmetics” to accentuate certain body parts with eye paints, oils, lotions, henna, fragrances, ointments, etc. [92]. This went on from era to era until Albert Kligman coined the term ‘cosmeceuticals’ in 1984. It is the marriage of both terms “cosmetics” and “pharmaceuticals” to define products that have cosmetic and therapeutical properties [93]. With the current trend pushing for more natural-based products, many cosmetic companies have begun marketing products that contain natural extracts obtained from the environment, for example, plants, animals, and volcanic soil [94].

### 6.1. Melatonin as a Cosmeceutical Product

Bringing our current subject of interest into context, products containing melatonin have been circulating the market for some time. Why melatonin? one might ask. This is because in the skin, melatonin is being generated naturally and has been found to have anti-aging and photoprotective properties [95,96,97,98,99]. Production of serotonin begins with L-tryptophan. This is then transformed into melatonin through enzymes generated in the epidermal and dermal sections of the skin [100]. As both serotonin and melatonin are hormones that regulate the central circadian rhythm, their presence and contribution as regulators of the skin’s circadian rhythm and homeostatic system were not surprising [101]. Aside from that, melatonin as an active ingredient in all forms of cosmetic treatment had been reported to be a global market net worth of 700 million USD just in the year 2018. It has even been projected to reach 2790 million USD by the year 2025 [102].

Diving into the photoprotective properties of melatonin, the application of melatonin to human dermal fibroblasts has been reported to stimulate *PER1* gene expression, showing its close relationship with the core clock genes [103]. Later, it was confirmed that melatonin is both an antioxidant and an antioxidant stimulant by nature [104]. Ex vivo human skin exposed to UVR recorded the ability of melatonin to inhibit UVR-induced DNA damage by increasing the activity of endogenous antioxidative enzymes [105]. Clinically, when topical melatonin was applied, participants exposed to sunlight were measured to have reduced erythema as compared to areas that were not applied with melatonin [106]. In addition, melatonin also metabolizes rapidly in the skin into metabolites such as 6-hydroxymelatonin and 5-methyoxytryptamine by the indolic pathway. It also forms *N*^1^-acetyl-*N*^2^-formyl-5-methoxykynuramine (AFMK) via the kynuric pathway enzymatically or non-enzymatically under the presence of UVB and free radicals. AFMK can then also be further metabolized to *N*^1^-acetyl-5-methoxykynuramine (AMK) [107]. More in-depth information on a more complete metabolism pathway of melatonin in the skin and how they help to maintain and build the epidermal barrier can be found in comprehensive reviews by Kim, et al. [108] and Slominski, et al. [109]. Nevertheless, these metabolites, that are produced during the reaction of melatonin with ROS, together scavenge ROS in a cascade of reactions [110]. By mitigating the production of ROS, it could then be expected that melatonin would have an impact on both the skin’s inflammatory and apoptotic pathways [111,112] as the activation of both pathways comes hand-in-hand after the elevation of UVR-induced ROS. Beyond that, with the suppression of cutaneous inflammation, collagen degradation would also be inhibited concomitantly as the production of MMPs is triggered by skin inflammatory agents [18,113]. Overall, the inhibition of this train of events will ultimately lead to delayed premature skin aging which is desired by cosmetic companies. However, as we age, levels of melatonin receptor 1 (MT-1) in our skin reduce, increasing our chances of UVR-induced photodamage [103]. Hence, besides the addition of melatonin in cosmetics, it is imperative to search for alternative active ingredients that can either boost the levels of MT-1 in aged skin or modify the skin’s circadian rhythm through other pathways.

### 6.2. Potential Use of Natural Products as a Modulator of the Circadian Rhythm

**Figure 4:**
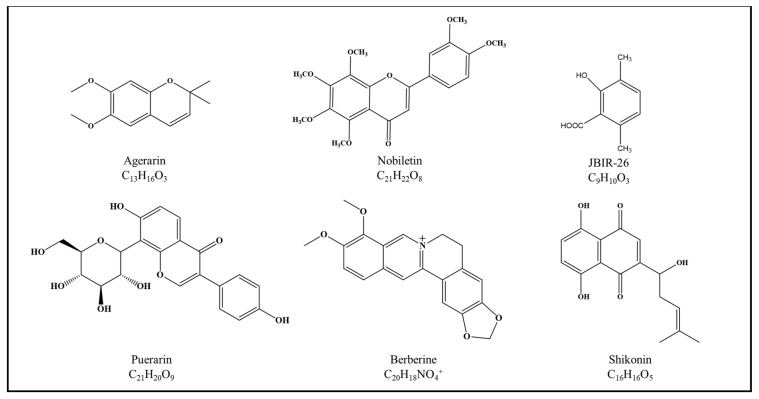
Chemical structure of agerarin, nobiletin, JBIR-26, puerarin, berberine, and shikonin.

Besides melatonin, there are various other biocompounds (Figure 4) and natural product extracts that can modulate the core clock expression directly or indirectly. Indirect pathways would be through opsins and, in one case, through microRNA (miRNA) expressions as depicted in Table 2. On top of their effect on opsins or core clock expressions, other bioproperties, such as anti-pigmentation, anti-inflammation, and wound healing, that are desirable in the development of cosmetics, were also reported. Nevertheless, no mechanistic studies depicting the connection between these bioproperties and core clock genes have yet been conducted for all aforementioned natural products. Even so, given the relationship between circadian rhythm and photoaging, one could hypothesize that there is a high possibility that these natural products might exhibit some of these bioproperties through their control over the core clock genes/proteins. On another note, one research stands out in which the compound, nobiletin, was shown to rescue rhythmicity in MDA-MB-231 cancer cells, changing their oncogenic features [114]. The implications of this study demonstrate that there is a potential where nobiletin can be used, instead, to reactivate rhythmicity in aged skin cells. Just as UVA was utilized by Pernodet, Dong, and Pelle [85] to reintroduce circadian rhythmicity in aged fibroblast cells, nobiletin, too, can also be used. With rhythmicity restored in aged skin cells, it is hypothesized that cells could once again regain their ability to withstand UVR photodamage. Incidentally, throughout this review, it was noted that no studies have reported the impact of UVR on RORs and Rev-erbα. Moreover, studies concerning the effect of natural products on these two proteins were far and few in between. As RORs and Rev-erbα are essentially part of the circadian rhythm, it would be worthwhile to look into the effect of UVR and natural products on their expression. Hence, this highlights another research gap that had yet to be explored in the world of cosmeceutical.

**Figure 5:**
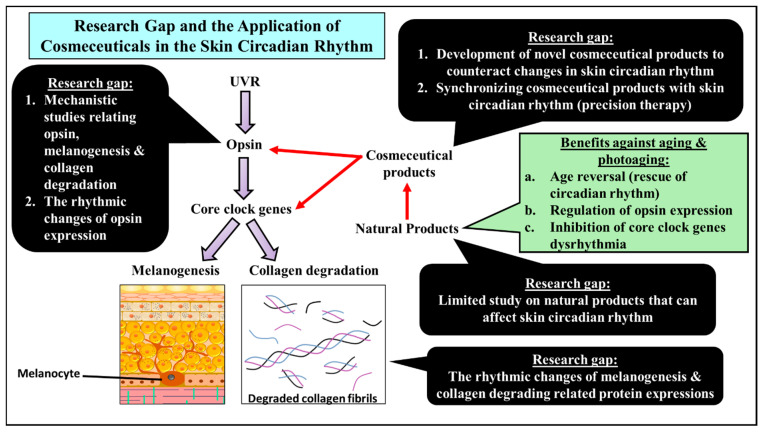
A summary of the research gap and the use of natural product-based cosmeceuticals in step with the skin’s circadian rhythm.

Ultimately, the use of natural products, in addition to melatonin, to modulate skin’s circadian rhythm is a completely new venture that had been overlooked. Not only could it aid in alleviating the symptoms of photoaging but, by actively reversing the loss of rhythmicity in aged skin cells, our skin’s robustness against UVR could be maintained or improved as we aged. Aside from that, timely use of these natural products as a cosmeceutical in sync with the skin’s rhythm could further enhance the effectiveness of their bioproperties on our skin, increasing their value to us. Research gaps that were mentioned in this review and the benefits of using natural product-based cosmeceuticals are summarized in Figure 5.

## 7. Conclusions

In short, exposure of our skin to UVR does affect our skin’s circadian rhythm, leading to the premature aging of our skin and in some cases, the development of skin cancer. However, as core clock gene expression changes periodically throughout the day, exposure at certain hours of the day proves to be more harmful than others. Therefore, applying “custom/specialized” natural product-based cosmeceutical at those specific hours could potentially aid in reversing the detrimental effect of UVR on our skin. Yet, there is still much to learn of the skin’s circadian rhythm. This includes the search for effective natural products that can rescue our skin cells from photoaging through their impact on the skin’s circadian rhythm. Hence, more research is still warranted in the field of skin circadian rhythm.

## Figures and Tables

**Table 1:** The significant changes in core clock genes after being irradiated with UVB. (NB-UVB: narrowband UVB; BB-UVB: Broadband UVB).

Type of UVR	Dose	Cell Type	Expression of Core Clock Gene and Protein after UVR	References
NB-UVB (311 nm)	688 J/cm^2^	Epidermal/dermal (Primary cell culture obtained from skin biopsies of skin phototypes II and III after irradiation)	↓ *CRY2* mRNA (24 h after exposure)	[25]
BB-UVB (280–320 nm)	5 mJ/cm^2^	HaCaT (Immortalized normal human keratinocyte cell line)	↓ *BMAL1* mRNA (immediate effect from 0 to 8 h); ↑ *BMAL1* mRNA (24 h after exposure); Marginal changes in BMAL1 protein after exposure	[26]
			↓ *CLOCK* mRNA (immediate effect from 0 to 8 h); *CLOCK* mRNA return to basal level (24 h after exposure); Marginal changes in CLOCK protein after exposure	
		HKC (primary human keratinocyte cells)	↓ *BMAL1* mRNA (immediate effect from 0 to 8 h); ↑ BMAL1 (24 h after exposure)	
			↓ *CLOCK* mRNA (immediate effect from 0 to 8 h); ↑ *CLOCK* mRNA (24 h after exposure);	
	10 mJ/cm^2^	Normal human keratinocyte(Primary cell culture from neonatal foreskin)	↓ *BMAL1* mRNA (immediate effect from 0 to 20 h)	[27]
			↓ *PER1* mRNA (from 0 to 12 h)	
			↓ *CLOCK* mRNA (from 0 to 24 h)	
	12.5 mJ/cm^2^	HaCaT	↓ *BMAL1* mRNA and protein (at 24 h)	[28]
	20 mJ/cm^2^	NHEK (Normal neonatal human epidermal keratinocytes)	↓ *CLOCK* mRNA and protein (Immediate effect after exposure)	[29]
			↓ *BMAL1* mRNA and protein (Immediate effect after exposure)	

**Table 2:** Natural products that were able to modulate opsins or core clock gene/protein expressions and their reported bioproperties in various studies that may contribute to the development of new cosmeceuticals.

Natural Product	Testing Model	Effect on Opsins or Core Clock Expression	Reported Bioproperties Potentially Beneficial in Cosmeceutical Development	References
Shikonin (isolated from *Lithospermum erythrorhizon* and *Arnebia euchroma*)	NIH3T3-derived stable cells transfected with BMAL1 promoter (NIH3T3 cells: immortalized embryonic fibroblast)	Shortens *BMAL1* transcriptional oscillation	Anti-cancer, anti-tumour, anti-inflammation, wound healing, anti-bacterial, anti-fungal	[115,116,117,118,119,120,121]
Nobiletin (polymethoxylated flavone from the skin of citrus fruits)	U2OS cells (bone osteosarcoma) MCF7 cells (breast adenocarcinoma) MDA-MB-231 cells (breast adenocarcinoma)	Subtle circadian alterations in U2OS and MCF7 Enhancement of circadian oscillation in MDA-MB-231 (rescue of rhythmicity)	Anti-inflammation, anti-tumour, antioxidant, anti-microbial, anti-pigmentation, anti-collagen degradation	[114,122,123,124,125,126,127]
	CLOCK^Δ19/+^-immortalized mice fibroblast cells Hepa1-6 cells (murine hepatoma) U2OS cells	Enhancement of amplitude and lengthen period of the *PER2::LucSV* reporter rhythm Modification of the expression of other core clock genes and proteins Direct binding to RORα and γ, inducing *BMAL1* promoter-driven luciferase reporter expression		
Berberine (Isolated from *Coptidis rhizoma*)	Bone marrow-derived macrophages from C57BL/6 mice HEK293 cells	Antagonization of Rev-erbα activity ↓ *BMAL1* expression	Anti-inflammation, anti-pigmentation, anti-collagen degradation, anti-microbial, anti-cancer	[128,129,130,131,132,133]
Puerarin (Isolated from *Puerariae radix*)	Hepa-1c1c7 cellsHEK293 cells	Antagonization of Rev-erbα activity ↑ *BMAL1* expression	Wound healing, anti-inflammation, anti-cancer Promotes melanogenesis *	[134,135,136,137,138]
JBIR-26 (derived from *Streptomyces* sp. AK-AH76)	NIH3T3 cells transfected with PER2 promoter	Lengthening of *PER2* transcriptional oscillation	Not available	[139]
Agerarin(Isolated from *Ageratum houstonianum*)	HaCaT cells	↑ *CLOCK* mRNA expression	Increases aquaporin 3 expression (skin moisturizing), anti-pigmentation, increases filaggrin involved in maintaining skin barrier	[140,141,142]
*Lepedeza capitata* extract	HaCaT cells	↑ *PER2* activity Lengthening of period of Per2 promoter activity	Wound healing, anti-collagen degradation	[70,143]
*Trichosanthes kirilowii* ethanolic extract	HaCaT cells	↓ *miR-142-3p* which inhibits BMAL1 expression	Anti-pigmentation, protection against UVB-induced DNA damage, anti-inflammation, anti-cancer	[28,144,145,146]
Fernblock^®^ (hydrophilic extract from *Polypodium leucotomos* leaves)	B16-F10 cells (mouse melanocyte)	↓ *OPN3* mRNA expression	Anti-pigmentation, Antioxidant, protection against UVR-induced DNA damage, anti-collagen degradation	[147,148,149]
*Centella asiatica* ethanolic extract	ARPE-19 cells (human retinal pigmented epithelium)	↑ OPN2 protein ↑ Opsin protein	Anti-pigmentation, anti-inflammation, increase skin hydration, anti-collagen degradation, anti-microbial, anti-fungal	[150,151,152,153,154,155]

* Reported bioproperties that could be used instead for the development of medicine for skin diseases, such as vitiligo.

## Data Availability

No new data were created or analyzed in this study. Data sharing is not applicable to this article.
